# Expression of OA1 limits the fusion of a subset of MVBs with lysosomes – a mechanism potentially involved in the initial biogenesis of melanosomes

**DOI:** 10.1242/jcs.128561

**Published:** 2013-11-15

**Authors:** Thomas Burgoyne, Rushee Jolly, Belen Martin-Martin, Miguel C. Seabra, Rosanna Piccirillo, Maria Vittoria Schiaffino, Clare E. Futter

**Affiliations:** 1 UCL Institute of Ophthalmology, 11–43 Bath Street, London EC1V 9EL, UK; 2 National Heart and Lung Institute, Imperial College London, London SW7 2AZ, UK; 3 IRCSS-Istituto di Ricerche Farmacologiche ″Mario Negri″, via La Masa, 19 20156 Milan, Italy

**Keywords:** Lysosomes, Multivesicular bodies, OA1

## Abstract

Multivesicular endosomes/bodies (MVBs) deliver proteins, such as activated EGF receptor (EGFR), to the lysosome for degradation, and, in pigmented cells, MVBs containing PMEL are an initial stage in melanosome biogenesis. The mechanisms regulating numbers and fate of different populations of MVB are unclear. Here, we focus on the role of the G-protein-coupled receptor OA1 (also known as GPR143), which is expressed exclusively in pigmented cells and mutations in which cause the most common type of ocular albinism. When exogenously expressing PMEL, HeLa cells have been shown to form MVBs resembling early stage melanosomes. To focus on the role of OA1 in the initial stages of melanosome biogenesis we take advantage of the absence of the later stages of melanosome maturation in HeLa cells to determine whether OA1 activity can regulate MVB number and fate. Expression of wild-type but not OA1 mutants carrying inactivating mutations or deletions causes MVB numbers to increase. Whereas OA1 expression has no effect on delivery of EGFR-containing MVBs to the lysosome, it inhibits the lysosomal delivery of PMEL and PMEL-containing MVBs accumulate. We propose that OA1 activity delays delivery of PMEL-containing MVBs to the lysosome to allow time for melanin synthesis and commitment to melanosome biogenesis.

## Introduction

The role of multivesicular bodies (MVBs) in the sorting of lysosomally targeted growth factor receptors, such as EGF receptor (EGFR), is well established. Lysosomally targeted EGFRs are sorted onto the intraluminal vesicles (ILVs) of MVBs, whereas recycling receptors, such as transferrin receptor remain on the perimeter membrane of the MVB from where they are recycled. When all the recycling proteins have been removed, the mature MVB can fuse with the lysosome and the contents are degraded (Futter et al., 1996). In more recent years, it has become clear that lysosomal fusion is not the only possible fate for MVBs. In pigmented cells MVBs can mature into melanosomes (Raposo et al., 2001) and in some cell types MVBs can fuse with the cell surface, releasing the ILVs into the extracellular space as exosomes (van Niel et al., 2006). The relationship between the MVBs with these different fates is not clear, but multiple populations of MVBs can exist within the same cell type. For example, activated EGFRs are trafficked in a separate population of MVBs to those that carry LBPA (White et al., 2006). Furthermore, multiple mechanisms for sorting cargo onto ILVs and for generating ILVs have been described. The sorting of EGFR onto ILVs depends on the endosomal sorting complexes required for transport (ESCRT) machinery (Doyotte et al., 2005; Razi and Futter, 2006). The sorting of the melanogenic-cell-specific protein PMEL onto ILVs is accompanied by proteolytic events that lead to the generation of fibrils within the immature melanosome upon which melanin is deposited (Berson et al., 2001), PMEL sorting to ILVs is ESCRT independent (Theos et al., 2006) but depends on the tetraspannin, CD63 (van Niel et al., 2011). In an oligodendroglial cell line, the sorting of the proteolipid protein onto ILVs that can subsequently be released as exosomes depends on the sphingomyelinase-dependent production of ceramide (Trajkovic et al., 2008). Although these different mechanisms of sorting and ILV formation have been described, it is not clear to what extent the different ILV formation mechanisms are segregated within different types of MVB or how the relative numbers of the different MVB subpopulations are regulated.

Our understanding of the regulation of lysosome number has recently been increased through the identification of the ‘CLEAR’ network of lysosomal genes, whose transcriptional upregulation causes an increase in lysosome number (Sardiello et al., 2009). Unlike lysosomes, which can be relatively long-lived, MVBs are not stable compartments and depend on membrane flux from the plasma membrane and early endosomes and are consumed by fusion with late endosomes/lysosomes. EGF stimulation increases not only the number of ILVs but also the number of MVBs, indicating that the biogenesis of at least the EGFR-containing subpopulation of MVBs can be regulated (White et al., 2006). Melanosome biogenesis is also subject to upregulation and downregulation; this occurs in a short window in embryonic life in retinal pigment epithelial cells followed by perinatal downregulation (Lopes et al., 2007), whereas, in melanocytes, melanosome biogenesis is upregulated following UV exposure (Imokawa, 2004). Whether upregulation of melanosome biogenesis is accompanied by upregulation of MVB biogenesis or diversion of MVBs from the lysosomal pathway is not clear.

OA1 (also known as GPR143) is a seven transmembrane protein that shares structural and functional homology with heterotrimeric G-protein-coupled receptors (GPCRs) and is expressed exclusively in melanogenic cells (Schiaffino et al., 1996; Sone and Orlow, 2007). Mutations in the OA1-encoding gene are responsible for the most common type of ocular albinism (type 1), in which patients exhibit hypopigmentation of the retina and iris, nystagmus and loss of visual acuity (King et al., 1995). In multiple systems OA1 behaves like a bona fide GPCR (Schiaffino et al., 1999; Innamorati et al., 2006; Staleva and Orlow, 2006) and some disease-causing mutations are in residues highly conserved in most GPCRs (d'Addio et al., 2000) suggesting that ocular albinism type1 is caused by a loss of GPCR activity. Loss of OA1 activity in patients and knockout mice leads to a reduced number of enlarged melanosomes (O'Donnell et al., 1976; Incerti et al., 2000), which also exhibit changes in their distribution (Palmisano et al., 2008). Reduced melanosome numbers precede the increase in melanosome size, indicating that there is a reduction in melanosome biogenesis, rather than simply enhanced melanosome fusion (Incerti et al., 2000). Unlike most GPCRs, which localise to the plasma membrane, OA1 localises to immature and mature melanosomes and late endosomes/lysosomes in pigmented cells, and, when expressed in cells lacking melanosomes, it localises to MVBs and lysosomes (Schiaffino et al., 1996; Samaraweera et al., 2001; Piccirillo et al., 2006; Gordiano et al., 2009). This localisation, together with the reduced melanosome number that occurs on loss of OA1, has led to the suggestion that OA1 might regulate MVB fate. Although melanosomes and lysosomes share constituents and a common origin, the relationship between the pathways is complex and the regulation of MVB fate (melanosome versus lysosome) is poorly understood. In cultured melanocytes, depletion of OA1 induces the formation of enlarged disorganised melanosomes that contain lysosomal as well as melanosomal constituents (Giordano et al., 2009). This suggests that OA1 might play a role in the early segregation of the melanosomal and lysosomal pathways that occurs at the level of early MVBs (stage 1 melanosomes) (Raposo et al., 2001). This is a comparatively short-lived stage in melanogenic cells, as delivery of melanin-synthesising enzymes and melanin deposition rapidly occurs, making the regulation of stage I melanosome number and fate difficult to quantify. Non-melanogenic cells have an equivalent stage to which melanosomal constituents, like PMEL, are targeted upon ectopic expression (Berson et al., 2001). Here, we take advantage of the absence of a melanogenesis pathway in HeLa cells to determine whether OA1 expression alone can modulate MVB number and fate. Furthermore, the absence of the machinery for melanosome biogenesis makes it possible to examine the direct effects of OA1 on lysosomal delivery. By comparing the effects of expression of wild-type and OA1 proteins carrying inactivating mutations, we show that expression of OA1 can regulate the numbers of a subset of MVBs through inhibition of lysosomal delivery.

## Results

### Overexpression of OA1 increases MVB and lysosome number

**Fig. 1.**
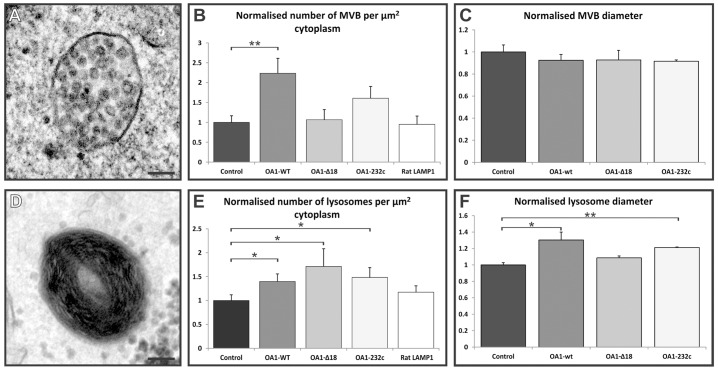
**Expression of wild-type OA1 in HeLa cells increases MVB and lysosome number.** HeLa cells were transiently transfected with OA1-wt, OA1 mutants or rat LAMP1 and processed for conventional electron microscopy. The numbers of MVBs per unit cytoplasm, and the diameter of MVBs (A–C) and lysosomes (D–F) were measured. A representative MVB and lysosome are shown in A and D. Results are means±s.e.m. of three experiments. *P<0.05, **P<0.01. There is a significant increase in the number of MVBs in HeLa cells expressing OA1-wt but not OA1 mutants, whereas there are increased lysosome numbers in cells expressing both OA1-wt and OA1 mutants. OA1 expression has no effect on MVB diameter but there is a significant increase in lysosome size on expression of OA1-wt and the OA1-232c mutant. Scale bars: 100 nm.

To determine the effect of overexpressing OA1 on the endocytic pathway, HeLa cells were transiently transfected with Myc-tagged OA1 wild-type (wt) and OA1 mutants, and the number of MVBs and lysosomes per µm^2^ of cytoplasm was analysed by electron microscopy. MVBs and lysosomes were distinguished on the basis of morphology; MVBs have one or more discrete ILVs (Fig. 1A) and lysosomes might contain ILVs but are also electron dense and contain irregular membrane whorls (Fig. 1D). Results were normalised to the average values determined for non-transfected controls. Expression of wild-type OA1 significantly increased the formation of both MVBs and lysosomes (Fig. 1B,E). Expression of OA1-232c (T232K), which contains a single missense mutation found in ocular albinism type 1 patients, and OA1-Δ18, which has a deletion of 18 amino acids within the i3 cytosolic loop region of OA1 responsible for its GPCR activity (Innamorati et al., 2006; Palmisano et al., 2008) did not cause a significant increase in MVBs, suggesting that the increased MVB number caused by overexpression of OA1-wt is dependent on OA1 activity. Expression of an unrelated GPCR, rhodopsin, did not affect MVB number (supplementary material Fig. S1). Overexpression of OA1 mutants that are inactive as GPCRs and expression of rhodopsin did cause some increase in lysosome formation, suggesting that this effect was independent of the GPCR activity of OA1. We therefore analysed the effects of overexpressing the rat lysosomal membrane protein LAMP1, which has no known GPCR activity or homology to OA1. Consistent with a role for OA1 activity in regulating MVB number, there was no effect of LAMP1 overexpression on MVB number but there was a small, although not significant, increase in lysosome number, consistent with the possibility that expression of a lysosomal membrane protein can increase lysosome number. Although OA1 expression increased MVB number, there was no significant increase in MVB size upon expression of any of the OA1 constructs tested (Fig. 1C). Increased lysosome size was observed upon expression of wild-type OA1 (Fig. 1F) and a smaller increase was observed upon expression of OA1-232c, but not OA1-Δ18, suggesting that OA1 activity might be required for increased lysosome size. It was not possible to determine which individual cells had been transfected in these experiments and thus for each construct the quantification represents a mixed population of expressing and non-expressing cells, although an equivalent expression frequency (∼40%) was obtained for all constructs. Therefore any observed effects of constructs on organelle number and size are likely to be an underestimation of the true effect of overexpressing each form of OA1.

**Fig. 2.**
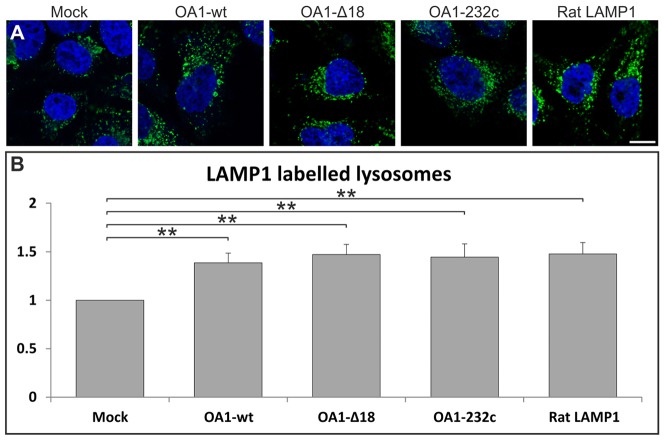
**Expression of lysosomal associated membrane proteins increases lysosome number.** (A) Representative confocal images showing punctate localisation of human LAMP1 in addition to nuclear DAPI staining (blue) in HeLa cells expressing either rat LAMP1 or various forms of OA1 (green). (B) Quantification of the number of LAMP1-positive punctae in the cells described in A. Results are means±s.e.m. of five experiments. **P<0.01. In HeLa cells expressing OA1, OA1 mutants or rat LAMP1 there is a significant increase in the number of LAMP1-positive punctae when compared to mock-transfected cells. Scale bar: 50 µm.

To determine whether the lysosomes induced by OA1 expression contained a bona fide lysosomal membrane protein, the number of LAMP1-positive punctae per cell in cells transfected with wild-type OA1, OA1 mutants or rat LAMP1 was quantified by immunofluorescence using an antibody specific for human LAMP1 that does not stain the expressed rat LAMP construct. Overexpression of OA1, OA1 mutants and the unrelated lysosomally targeted rat LAMP1 construct increased the number of LAMP1-positive punctae per cell (Fig. 2).

Although LAMP1 is a lysosomal membrane protein, in some cell types it can also be found in earlier endocytic structures, raising the possibility that some of the LAMP1-positive structures quantified by immunofluorescence could be MVBs. Cryo-immunoelectron microscopy (supplementary material Fig. S2A) revealed that lysosomes contained an ∼2-fold higher density of LAMP1 than do MVBs (supplementary material Fig. S2B). OA1 is also distributed between MVBs and lysosomes. The same number (∼70%) of OA1-positive structures stain for LAMP1 by immunofluorescence and have the morphology of lysosomes as determined by electron microscopy studies (supplementary material Fig. S2C–E). We conclude therefore that, despite the presence of small amounts of LAMP1 on MVBs, the density is insufficient under the conditions of our immunofluorescence experiments for them to be detected; hence, our quantitative LAMP1 immunofluorescence predominantly measures lysosomes.

The use of LAMP1 as a lysosomal marker to determine effects of OA1 expression on lysosome biogenesis and delivery relies on OA1 expression not altering the distribution of LAMP1. The density of LAMP1 immunostaining on MVBs and lysosomes was therefore compared in OA1-expressing and control cells. LAMP1 density was unaffected by OA1 expression (supplementary material Fig. 2B), further justifying its use as a lysosomal marker in analysis of the effects of OA1 activity on lysosome biogenesis and delivery.

### OA1-wt and mutant OA1-Δ18 and OA1-232c localise to MVBs and lysosomes

**Fig. 3.**
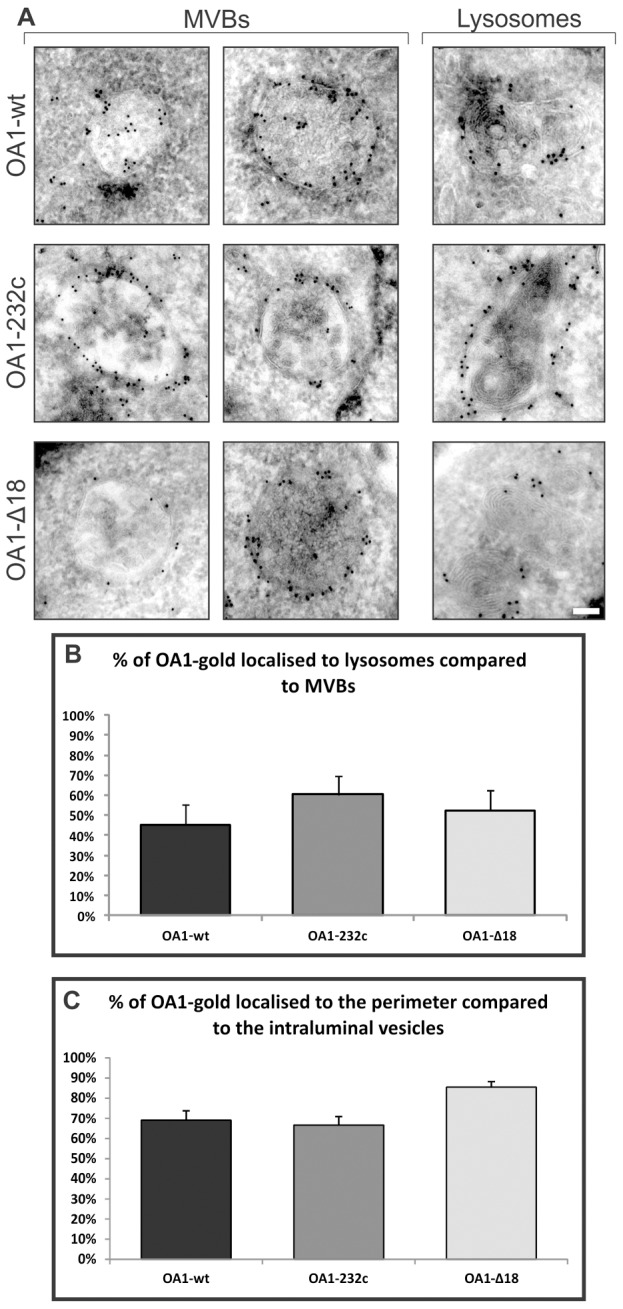
**Wild-type OA1 and OA1 with a point-mutation (232c) or deletion of 18 amino acids (Δ18) in the i3 cytosolic loop localises to MVBs and lysosomes.** (A) HeLa cells were transfected with Myc-tagged OA1 and OA1 mutants and embedded for cryo-immunoelectron microscopy. Thawed cryosections were stained with anti-Myc antibody. OA1-wt and mutants localised to morphologically similar MVBs and lysosomes. (B) The number of gold particles (gold-labelled anti-Myc antibody) in lysosomes were quantified and compared with numbers in MVBs. A smaller percentage of OA1-wt localised to lysosomes, compared with the inactive OA1 mutants. (C) The number of gold particles localising to the perimeter membrane and the ILVs of MVBs were quantified. More OA1-Δ18 was found on the perimeter membrane than OA1-wt or 232c. Scale bar: 200 nm.

Some disease-causing missense mutations in OA1 cause it to fail to exit the ER, whereas other OA1 mutants localise to the endocytic/melanosomal pathway like the wild-type protein (d'Addio et al., 2000). We selected the Δ18 and 232c mutants of OA1 because they had previously been shown to localise to the endocytic pathway. However this did not preclude the possibility of subtle changes in the localisation of these mutant proteins compared to the wild type. We therefore analysed the localisation of OA1-wt and the mutants by cryo-immunoelectron microscopy. Immuno-gold labelling of samples using an antibody to the Myc epitope showed that the majority of wild-type and mutant forms of OA1 localised to MVBs and lysosomes and the morphology of the labelled organelles appeared very similar for each form of OA1 (Fig. 3A). Quantification of the relative distribution of OA1 between MVBs and lysosomes did, however, reveal differences in the distribution of wild-type and mutant proteins. Although, as described above, ∼70% of wt-OA1-positive structures are lysosomes, quantification of the density of OA1 labelling on MVBs versus lysosomes revealed that less than 50% of the gold particles labelling OA1 were present on lysosomes (Fig. 3B). There was an increased amount of Δ18 and 232c mutants of OA1 present on lysosomes, suggesting enhanced delivery of these mutants to lysosomes (Fig. 3B). Quantification of the distribution of the gold particles labelling OA1 on the perimeter membrane of the MVB versus the intraluminal vesicles revealed that ∼70% of OA1-wt and the 232c mutant remained on the perimeter membrane. An increased percentage of the Δ18 mutant remained on the perimeter membrane, suggesting that this mutant was deficient in targeting to the intraluminal vesicles (Fig. 3C). Together, these findings indicate that the mutant forms of OA1 are trafficked to the same organelles as the wild-type protein, despite some differences in their relative distributions. Therefore the disease phenotype that arises from these mutations within OA1 probably results from a reduced/lack of interaction with a single or multiple regulatory substrate(s) within the i3 OA1 domain.

### OA1 does not affect fusion of EGFR-containing MVBs with the lysosome

**Fig. 4.**
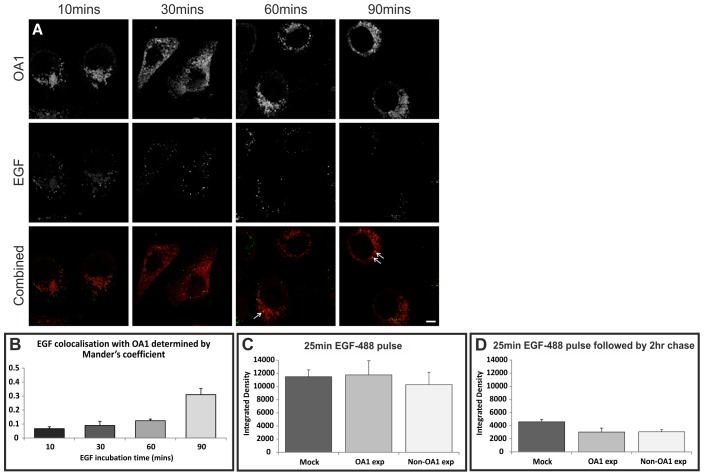
**OA1 expression does not affect delivery of EGF to the lysosome.** (A) Confocal images of HeLa cells pulsed with EGF-488 for 10, 30, 60 and 90 minutes. (B) There was very little colocalisation between EGF and OA1 until 60–90 minutes when EGF reaches lysosomes. (C) OA1 expression had no effect on the uptake of EGF after pulsing HeLa cells for 25 minutes with EGF-488. (D) After chasing with unlabelled EGF there was a reduction in EGF-488 fluorescence which was unaffected by OA1 overexpression. Results are means±s.e.m. of three experiments. Scale bar: 50 µm.

EGF is trafficked in a subset of MVBs whose numbers increase upon EGF stimulation. To determine whether OA1 is trafficked in the same MVBs as EGF, colocalisation of EGF–Alexa-Fluor-488 (EGF-488) with OA1-wt was measured over time. After 25 minutes of EGF stimulation, when the majority of EGF is in MVBs, EGF was predominantly localised to a different subset of endosomes to those containing OA1 (Fig. 4A,B). However, after incubating HeLa cells for at least 60 minutes with EGF-488, when a substantial amount of EGF has reached lysosomes, there was increased colocalisaton of EGF with OA1. Thus, EGF and OA1 appear to be trafficked in largely separate MVBs but meet in lysosomes. As we had previously found that LBPA is also trafficked in largely separate MVBs from those that carry EGF but that EGF and LBPA meet in the lysosome (White et al., 2006), we investigated colocalisation between OA1 and LBPA and found greatly increased colocalisation between OA1 and LBPA, compared with that between OA1 and EGF internalised for 25 minutes (supplementary material Fig. S3).

Increased numbers of MVBs could arise from an upregulation of MVB formation or reduced MVB consumption. In HeLa cells lacking a melanogenic pathway, the primary route for MVB consumption is fusion with the lysosome. EGF degradation depends on fusion of EGFR-containing MVBs with lysosomes. As described above OA1 and EGFR appear to be trafficked in separate populations of MVBs but meet in the lysosome. To determine whether there is a general inhibition of MVB–lysosome fusion by OA1 activity the effect of OA1 expression on the uptake and rate of EGF-488 degradation was determined. Expression of OA1 in HeLa cells had no significant effect on the uptake of EGF during a 25-minute pulse and, after a 2-hour chase, there was substantial loss of EGF-488 that was not significantly affected by expression of OA1 (Fig. 4C,D). Previous studies using this assay have demonstrated that loss of EGF-488 signal with time is prevented by pre-incubation of the cells with the protease inhibitor, leupeptin and under these conditions EGF accumulates in lysosomes, indicating that loss of EGF-488 signal represents lysosomal degradation (Eden et al., 2010). This suggests that OA1 expression has no effect on fusion of EGFR-containing MVBs with the lysosome.

### OA1 expression inhibits the delivery of fluid-phase probes to lysosomes

**Fig. 5.**
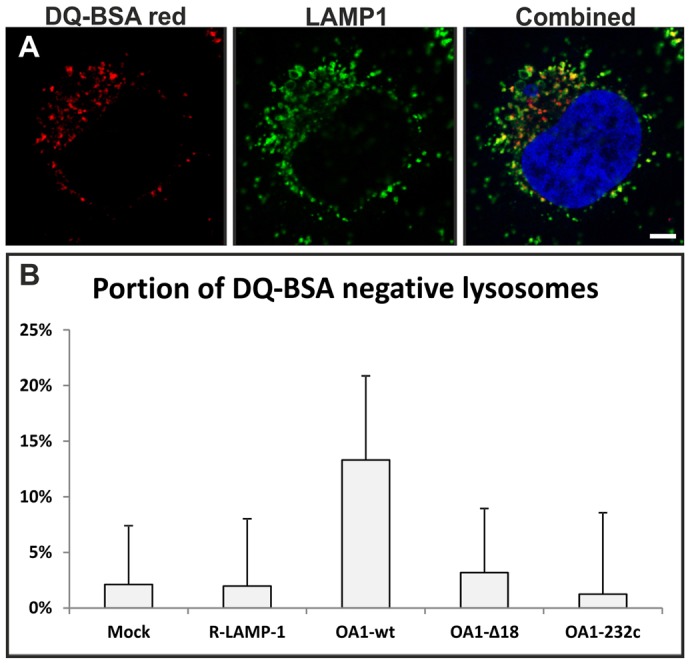
**Expression of OA1 reduces delivery of active DQ-BSA, the fluid-phase marker to the lysosomes.** HeLa cells transfected with Myc-tagged OA1 and OA1 mutants were subjected to a 2-hour pulse of DQ-BSA, followed by a 4-hour chase. (A) A representative confocal image of a HeLa cell fluorescently labelled with the active lysosomal marker DQ-BSA (red), LAMP1 (green) or a combined image including nuclear DAPI staining (blue). (B) Quantification of the subpopulation of DQ-BSA-negative lysosomes after DQ-BSA incubation of HeLa cells transfected with the indicated protein. Overexpression of wild-type OA1 increases the portion of DQ-BSA-negative lysosomes. Results are means±s.e.m. of five experiments. Scale bar: 20 µm.

**Fig. 6.**
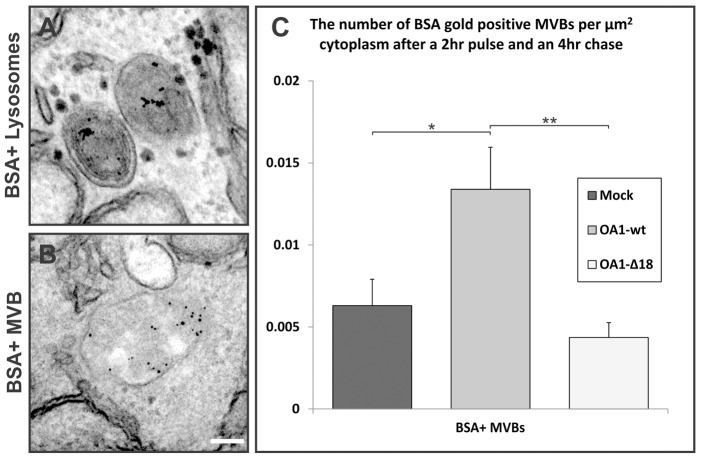
**Expression of OA1 increases the number of the BSA-gold positive MVBs after chasing to lysosomes.** HeLa cells transfected with Myc-tagged OA1 were subjected to a 2-hour pulse of DQ-BSA, followed by a 4-hour chase. (A) Representative electron micrograph image of lysosomes containing BSA–gold. (B) Representative electron micrograph image of MVBs containing BSA–gold. (C) Quantification of BSA–gold-positive MVBs in the cytoplasm. Results are means±s.e.m. of five experiments. *P<0.05, ** *P*<0.01. There are significantly increased numbers of MVBs containing gold in HeLa cells expressing OA1 compared to mock- and OA1-Δ18-transfected cells. Scale bar: 100 nm.

As OA1 was found to associate with a different subset of endocytic organelles to those containing EGF and EGFR, the rate of delivery of OA1-containing endosomes/MVBs to lysosomes could not be determined using EGF-488 alone. To determine whether the delivery or fusion to lysosomes was altered for OA1-containing MVBs, two experiments were performed using the fluid-phase marker BSA to follow the delivery of all populations of endosomes to lysosomes. In confocal experiments DQ-BSA was used as it only emits (at ∼515 nm wavelength) when hydrolysed in active lysosomes (Fig. 5) and for electron microscopy BSA tagged to 5-nm gold was used (Fig. 6). For both experiments the BSA markers were pulsed for 2 hours to allow adequate uptake before chasing with non-conjugated BSA for 4 hours to allow movement of the marker to the lysosome. The time periods used in this experiment were based on previous research showing that this is adequate to chase a fluid-phase marker from endosomes and for it to accumulate in lysosomes (Futter et al., 1996). An indicator of restricted or inhibited delivery to lysosomes is reduced DQ-BSA signal in LAMP1 positive lysosomes and increased numbers of MVBs positive for 5-nm-gold-tagged BSA. In non-transfected HeLa cells and cells transfected with mutant forms of OA1, almost all LAMP1-positive lysosomes were also positive for DQ-BSA signal, whereas in cells transfected with OA1-wt, a proportion of LAMP1-positive lysosomes did not contain the DQ-BSA signal (Fig. 5B). This could reflect reduced delivery of DQ-BSA to lysosomes or reduced activity of lysosomes, rendering them less efficient at proteolytic activation of the DQ-BSA signal. We therefore investigated the effect of OA1 expression on delivery of BSA-gold to lysosomes and found that, although the majority of lysosomes contained BSA–gold whether or not the cells had been transfected with OA1, there was a significant increase in the number of MVBs containing 5-nm-gold-tagged BSA gold in OA1-wt-transfected cells compared to mock-transfected cells and cells transfected with the OA1-Δ18 mutant (Fig. 6C). Taken together, the two experiments provide evidence for a reduction or slowdown of delivery of fluid-phase markers to lysosomes.

### OA1 expression inhibits delivery of PMEL to lysosomes

**Fig. 7.**
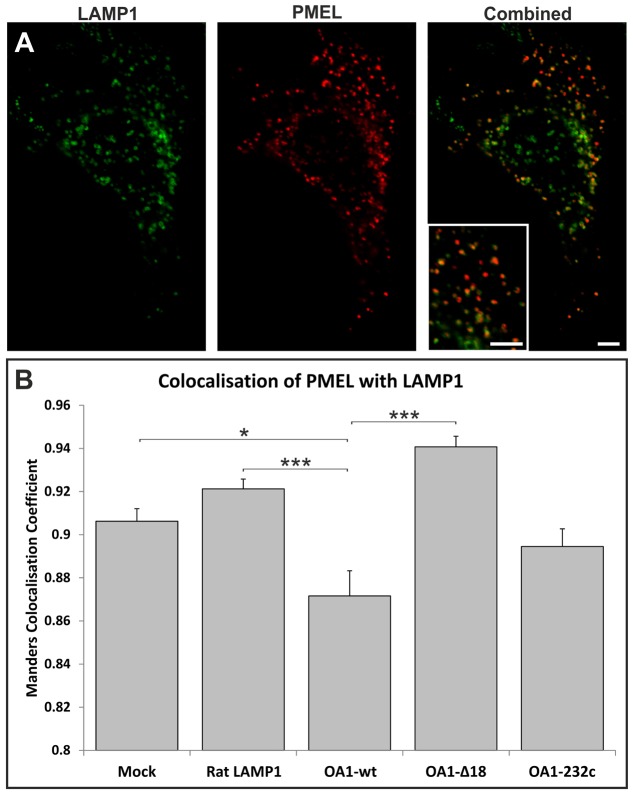
**The delivery of the melanosome-associated protein PMEL to lysosomes is reduced in OA1-expressing HeLa cells.** (A) A representative confocal image of a HeLa cell overexpressing PMEL fluorescently stained for PMEL (red) and LAMP1 (green). (B) Quantification of PMEL and LAMP1colocalisation using the Manders coefficient. Results are means±s.e.m. of three experiments. *P<0.05, ***P<0.001. Expression of OA1-wt significantly reduced PMEL and LAMP1 colocalisation compared to mock-, OA1-232c- and OA1-Δ18-transfected cells. Therefore, only the wild-type form of OA1 is able to restrict delivery of PMEL to lysosomes. Scale bars: 20 µm.

**Fig. 8.**
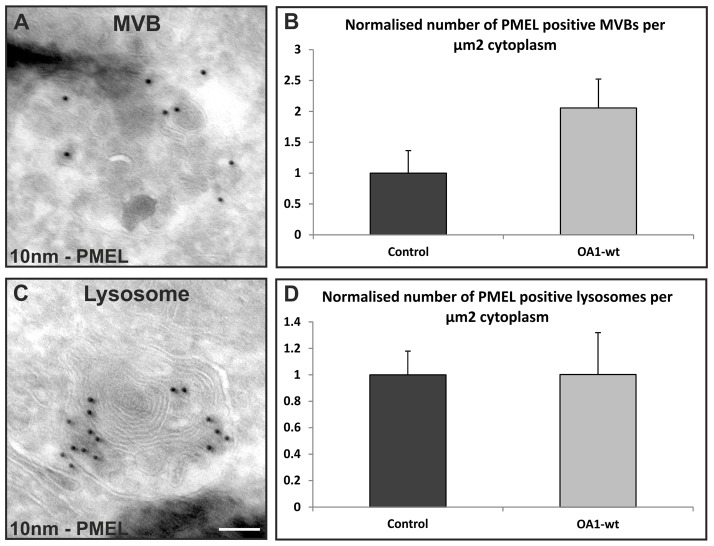
**Expression of wild-type OA1 in HeLa cells increases the number of PMEL-positive MVBs.** HeLa cells were transiently transfected with either PMEL or a combination of PMEL and OA1-wt, and processed for cryo-immunoelectron microscopy. Approximately 93% of cells expressing PMEL also expressed OA1 when transfecting with both constructs, as determined by parallel immunofluorescence. (A–C) Cryosections were stained with PMEL antibody and the number of PMEL-positive MVBs and lysosomes were measured. (B) Cells expressing OA1-wt had more PMEL-positive MVBs per unit of cytoplasm. (C) The number of PMEL-positive lysosomes was not affected by OA1-wt expression. Scale bar: 100 nm.

Using BSA as a fluid-phase marker does not allow the distinction between different populations of MVBs. To identify a cargo for the MVBs that accumulate upon OA1 expression HeLa cells were transfected with PMEL. PMEL was an attractive potential cargo because it localises to MVBs both in melanogenic cells and when expressed in non-melanogenic cells. It is required for normal melanosome biogenesis and has previously been shown to colocalise with OA1 in melanogenic cells. We found that PMEL localises to both MVBs and LAMP1-positive lysosomes when expressed in HeLa cells (Figs 7 and 8). In common with OA1, PMEL shows only limited colocalisation with EGF internalised for 25 minutes but shows extensive co-staining with OA1 when the two proteins are co-expressed (supplementary material Fig. S3). This indicates that, in HeLa cells, PMEL is largely trafficked to the lysosome in the same subpopulation of MVBs as OA1, which is distinct from the subpopulation that carries the majority of the EGFR. We reasoned that if OA1 induces a reduction or slowdown in fusion between PMEL-containing MVBs and lysosomes then there should be reduced colocalisation of PMEL with the lysosomal marker LAMP1 when OA1 is co-expressed. Indeed this was found to be the case in HeLa cells overexpressing OA1-wt; less PMEL colocalised with LAMP1 in these cells than in HeLa cells expressing mutant forms of OA1, the LAMP1 construct or in non-transfected cells (Fig. 7). Similarly expression of OA1-wt reduced the co-staining of co-expressed PMEL with lysotracker (supplementary material Fig. S4). This suggests that OA1 activity inhibits the fusion of a subset of MVBs that contain expressed PMEL with the lysosome. This would be expected to cause PMEL-containing MVBs to accumulate in OA1-expressing cells and, hence, cryo-immunoelectron microscopy was performed on mock-transfected and OA1-wt-transfected cells and the number of PMEL-positive MVBs per unit of cytoplasm quantified. As shown in Fig. 8, in OA1-transfected cells there is a more than 2-fold increase in the number of PMEL-positive MVBs compared with the number in control cells.

## Discussion

### Regulation of lysosome number by overexpression of lysosomal membrane proteins

We found that expression of two unrelated lysosomal membrane proteins, LAMP1, which is a single transmembrane domain protein with a large heavily glycosylated luminal domain, and OA1, which is a seven transmembrane domain GPCR, both led to an increase in lysosome number. That increased numbers of lysosomes can be caused by mutant forms of OA1 that do not induce detectable increases in MVB number suggests that the increased lysosome numbers are not simply due to increased membrane flux to the lysosome but are a response to the presence of increased resident protein copy number within the lysosomal membrane. This upregulation of lysosome number was evident within 24 hours of transfection with the lysosomal protein and represents an interesting example of how increasing membrane cargo increases the size of a compartment. The molecular detail of how lysosome number can be regulated by lysosomal content is beginning to be resolved. The Rag GTPase senses lysosomal amino acids, and regulates the activity of the mammalian target of rapamycin (mTOR) complex on the lysosomal membrane, which, in turn, regulates the starvation- and stress-induced nuclear translocation of transcription factor EB (TFEB) and the subsequent transcription of lysosomal genes (Settembre et al., 2012). Our data suggests that increased expression of lysosomal membrane proteins can also be sensed, although the mechanism of sensing is unclear.

OA1 expression caused an increase in lysosome size, consistent with previous data showing an increase in the diameter of LAMP2-positive compartments upon OA1 expression in Cos cells (Shen et al., 2001). Interestingly the increased lysosome number observed on expression of lysosomally targeted constructs was not inevitably accompanied by an increase in lysosome size because expression of the inactive mutant OA1-Δ18 caused an increase in lysosome number without an increase in size. Paradoxically OA1 activity acts in melanogenic cells to limit melanosome size as loss of OA1 causes formation of enlarged melanosomes, suggesting that effects of OA1 on lysosome size might reflect a different function of OA1 to that that regulates melanosome size. OA1 expression has previously been shown to modulate the distribution of mannose-6-phosphate receptor (Shen et al., 2001). Therefore, defects in lysosomal retrieval pathways could lead to lysosome enlargement. Alternatively delayed delivery of degradative enzymes via MVB–lysosome fusion could lead to reduced degradative capacity and consequent lysosomal enlargement.

### Regulation of MVB number by OA1 activity

The demonstration of the upregulation of MVB number by OA1-wt but not inactive OA1 mutants in HeLa cells demonstrates that OA1 is not only recognised by trafficking machinery but also retains some activity when expressed in non-melanogenic cells. The only previously described ligand for OA1 is L-DOPA (Lopez et al., 2008), which is generated by tyrosinase, an enzyme not expressed in HeLa cells. This suggests that the specific effects of OA1-wt in HeLa cells might depend either on the presence of alternative ligands able to activate the receptor or on its considerable constitutive activity (Innamorati et al., 2006). Furthermore, the increase in MVB number as a result of OA1 activity occurs without expressing MART1, which has previously been shown to stabilise OA1 (Giordano et al., 2009).

We have previously shown that signalling from activated EGFR causes an increase in the number of EGFR-containing MVBs. Here, we show that increased MVB number can also be caused by signalling from OA1. As MVBs are not stable entities, the regulation of their number is complex and represents a balance between their biogenesis and their fusion with their target membrane. In this study we show that, unlike EGFR signalling from the endosome, which is likely to promote lysosomal delivery, signalling from OA1 at the level of the endosome inhibits lysosomal delivery, leading to the accumulation of a subset of MVBs. We cannot exclude the possibility that OA1 also upregulates MVB biogenesis in HeLa cells. The MVBs that accumulate upon OA1 expression do not appear to be the same MVBs that deliver endocytosed EGF to the lysosome, as OA1 expression neither inhibits the lysosomal delivery of EGF nor localises to EGFR-containing MVBs. In contrast, when PMEL is expressed in conjunction with OA1, to identify MVBs that in melanogenic cells could become melanosomes, PMEL is targeted to the MVBs that accumulate upon OA1 expression. A simple interpretation of these data would be that EGFR signalling and OA1 signalling upregulate the numbers of separate populations of MVBs, EGF stimulation promoting the formation of an EGFR-containing subset, and OA1 activity inhibiting the lysosomal delivery of a different subset of MVBs. EGFR is sorted onto the ILVs of MVBs for subsequent lysosomal delivery by the ESCRT machinery, whereas PMEL utilises ESCRT-independent machinery for sorting onto ILVs. However ESCRT-mediated ILV formation and the formation of PMEL-containing ILVs are unlikely to occur in entirely separate MVBs as the C-terminal fragment of PMEL that remains after proteolytic cleavage is delivered to lysosomes in an ESCRT-dependent manner, suggesting that ESCRT-dependent and ESCRT-independent budding can occur on contiguous membranes (van Niel et al., 2011). Furthermore, OA1 itself has been shown to be capable of undergoing ubiquitylation and ESCRT-dependent sorting onto ILVs (Giordano et al., 2011). It is possible that the ESCRT machinery plays a role in the segregation of ESCRT-dependent lysosomally directed cargos from those that have other destinations, such as melanosomes, and that ubiquitylation of OA1 regulates its activity by removing it from immature melanosomes. The inability of the inactive OA1 mutants to increase MVB number was not owing to increased targeting to ILVs because wild-type OA1 and the patient mutation (232c) were targeted to ILVs with equal efficiency, and the OA1 mutation carrying an 18 amino acid deletion was less efficiently targeted to ILVs. It is possible that this deletion affects OA1 ubiquitylation.

We have shown that delivery of a subset of cargo to lysosomes is inhibited upon OA1 expression and that this inhibition is accompanied by an accumulation of MVBs. MVB cargo is delivered to lysosomes either by direct fusion (Futter et al., 1996) or by a ‘kiss and run’ process (Bright et al., 2005). The increased MVB number upon OA1 expression suggests that OA1 delays MVB–lysosome fusion. This is a process that depends on the Rab5 to Rab7 switch, the Rab7-dependent recruitment of the HOPs complex and a VAMP7-containing trans-SNARE complex (Luzio et al., 2010). OA1 could regulate one or more components of this machinery. Alternatively OA1 could delay MVB–lysosome fusion through effects on MVB or lysosome motility. This seems unlikely, however, as OA1 activity favours accumulation of melanosomes in the perinuclear region (Palmisano et al., 2008), which would be the type of movement likely to favour MVB–lysosome fusion. Alternatively OA1 expression could affect luminal pH of the MVBs to which it is localised and, thereby, inhibit MVB–lysosome fusion.

What is the purpose of delayed lysosomal fusion induced by OA1 expression? Delivery of PMEL to MVBs does not require the presence of OA1 as PMEL is targeted to MVBs in HeLa cells in the absence of OA1 (Berson et al., 2001). Delivery of PMEL is a comparatively early event in melanosome biogenesis. Delivery of the melanin-synthesising enzymes to maturing melanosomes is a later event that allows the deposition of melanin on pre-formed PMEL-dependent striations (Raposo et al., 2001). Delay in lysosomal fusion might allow time for delivery of melanin-synthesising enzymes and subsequent melanin deposition before lysosomal fusion. This could be of particular importance given the parallel role for OA1 in the regulation of melanosome maturation through the retention of immature melanosomes in the cell centre (Palmisano et al., 2008) bringing them into close proximity with lysosomes.

Lysosomal delivery of OA1 and PMEL was not completely blocked in HeLa cells given that some BSA–gold, expressed PMEL and OA1 could reach lysosomes in OA1-expressing cells. In melanogenic cells, the deposition of melanin might further inhibit lysosomal delivery or render the melanosome resistant to the effects of lysosomal fusion. Consistent with this hypothesis is our previous demonstration that inhibition of melanin deposition within melanosomes in RPE cells, either through inhibition of delivery of melanin-synthesising enzymes or through tyrosinase deletion, results in instability of immature melanosomes, which are then lost postnatally (Lopes et al., 2007). In melanogenic cells, OA1 deletion would be expected to enhance the fusion of PMEL-containing MVBs with lysosomes. This is difficult to measure directly but Giordano et al. (Giordano et al., 2009) showed that deletion of OA1 in melanocytes resulted in increased mixing of melanosome and lysosome markers, consistent with an enhanced fusion of immature melanosomes with lysosomes. This impaired, though did not prevent, melanin deposition.

Roles for OA1 in regulating melanosome number, size and transport have been identified through studies of melanogenic cells. To what extent these are independent functions remains unclear. The use of non-melanogenic HeLa cells in the present study has allowed the identification of a role for OA1 in the negative regulation of a specific transport step, namely MVB–lysosome fusion. The machinery recognising targeting signals of melanogenic proteins is conserved in non-melanogenic cells (Vijayasaradhi et al., 1995; Höning et al., 1998; Blagoveshchenskaya et al., 1999; Calvo et al., 1999; Simmen et al., 1999, Berson et al., 2001, Shen et al., 2001), and HeLa cells have MVBs equivalent to an early stage melanosome that carries the machinery necessary for sorting and proteolytic processing of PMEL and fibril formation (Berson et al., 2001; Theos et al., 2006). It seems reasonable to assume, therefore, that the delay in lysosome fusion that we have observed in HeLa cells would be conserved in melanogenic cells. Identifying the downstream effectors in HeLa cells that mediate the effects of OA1 on MVB number could provide targets that could be manipulated to determine the relationship between the different roles of OA1 in melanogenic cells and their relative importance in the prevention of ocular albinism.

## Materials and Methods

### Transfection

The OA1, OA1-232, OA1-Δ18 and rat LAMP1 constructs were generated as previously described (Palmisano et al., 2008). The OA1 constructs were cloned into pcDNA 3.1/Myc-His plasmids to allow comparable expression levels of each construct. The PMEL construct was prepared by Michael Marks at the University of Pennsylvania (Berson et al., 2001). HeLa cells were transfected using Lipofectamine 2000 reagent (Invitrogen) following the manufacturer's guidelines, for 48 hours with OA1–Myc, OA1-232–Myc, OA1-Δ18–Myc, PMEL or LAMP1. Before undertaking any experiments, the numbers of cells expressing each construct was determined by immunofluorescence using anti-Myc antibody to ensure equivalent transfection efficiencies.

### Electron microscopy

BSA was coupled to 5-nm colloidal gold as previously described (Slot and Geuze, 1985). After the pre-incubations indicated in the text, HeLa cells cultured on 3-cm dishes were fixed in 2% paraformaldehyde/2% glutaraldehyde followed by 1% osmium tetroxide/1% potassium ferrocyanide. Cells were dehydrated using increasing concentrations of ethanol and were then removed from plastic dishes using propylene oxide. Cells were recovered by centrifugation and cell pellets were then embedded in epon resin. Sections (70-nm thick) were cut through the full thickness of the pellet and examined using a Joel 1010 TEM. MVBs and lysosomes were counted in random sections. Vacuoles greater than 200 nm in diameter and having one or more ILVs in random sections and no irregular membranes were classified as MVBs, and electron-dense vacuoles greater than 200 nm in diameter containing irregular membranes were classified as lysosomes. At least 20 cells were quantified for each sample and the area of cytoplasm was measured for each cell section using ImageJ. Organelle diameters were measured using ImageJ, taking the mean of the longest and shortest diameter of each organelle.

### Cryo-electron microscopy

HeLa cells were prepared for cryo-immuno-electron microscopy by fixing with 4% paraformaldehyde in 0.1 M phosphate buffer at pH 7.4 and pelleting in 12% gelatin. Subsequently, after infusion with 2.3 M sucrose, 80-nm sections were cut at −120°C and collected in 1∶1 2.3 M sucrose/2% methylcellulose. The sections were immuno-labelled by incubating with anti-Myc (Millipore), anti-PMEL (HMB45 from Dako) or anti-LAMP1 (DSHB at the University of Iowa) antibodies and 10-nm-gold-tagged protein A (UMC Utrecht) as described previously (Slot et al., 1991).

### Fluorescence

HeLa cells were transfected for 48 hours with OA1–Myc, OA1-232–Myc, OA1-Δ18–Myc, PMEL or LAMP1. In some incidences cells were incubated with either (1) DQ-BSA red (Invitrogen) for 2 hours before unconjugated BSA for 4 hours or (2) EGF-488 for 25 minutes followed by unlabelled EGF for 2 hours. The cells were fixed in 2% paraformaldehyde and labelling using anti-Myc (Millipore and Abcam), HMB45 (Dako), ly1c6 (Enzo Life Sciences), LAMP1 (DSHB at the University of Iowa), CD63 (Santa Cruz Biotechnology) and LBPA (from J. Gruenberg, University of Geneva) antibodies. Image acquisition was performed on a Leica SP2 confocal, using the FITC and TRITC channels. To quantify the number of stained organelles in confocal slices and to measure colocalisation using the Manders' coefficient, ImageJ was used. Student's *t*-tests were performed to determine statistical significance.

## Supplementary Material

Supplementary Material
