# Supplementary material for: Expression of OA1 limits the fusion of a subset of MVBs with lysosomes – a mechanism potentially involved in the initial biogenesis of melanosomes
Source: J Cell Sci. 2013 Nov 15;126(22):5143–52. doi: 10.1242/jcs.128561 (PMC3828590; doi:10.1242/jcs.128561)
Supplement: Supplementary Material [file supp_126_22_5143__index.html]

Expression of OA1 limits the fusion of a subset of MVBs with lysosomes – a mechanism potentially involved in the initial biogenesis of melanosomes — Supplementary Material 

# Expression of OA1 limits the fusion of a subset of MVBs with lysosomes – a mechanism potentially involved in the initial biogenesis of melanosomes

## JCS128561 Supplementary Material

**Files in this Data Supplement:**

- **Supplementary Material PDF**
