# Supplementary material for: Expression of OA1 limits the fusion of a subset of MVBs with lysosomes – a mechanism potentially involved in the initial biogenesis of melanosomes
Source: J Cell Sci. 2013 Nov 15;126(22):5143–52. doi: 10.1242/jcs.128561 (PMC3828590; doi:10.1242/jcs.128561)

Supplementary Figure Legends

Supplementary Figure 1. Over-expression of rhodopsin increases the number of lysosomes. (A-B) When over-expressing rhodopsin there is no increase in the number of MVBs but there is an increase in the number lysosomes per μm^2 cytoplasm.

Supplementary Figure 2. LAMP1 predominantly stains lysosomes by immunofluorescence in the presence or absence of OA1 expression. (A) HeLa cells transfected with myc-tagged OA1 were embedded for cryo-immunoEM and stained with anti-myc and anti-LAMP1. (B) Lysosomes contained approximately twice the amount of LAMP1 staining compared to MVBs and OA1 expression did not affect the relative amounts of LAMP1 in MVBs and lysosomes. (C) HeLa cells expressing myc-tagged OA1 were stained for myc and LAMP1 and colocalising punctae were quantified. (D) HeLa cells expressing myc-tagged OA1 were prepared for cyro-immunoEM and stained for myc. OA1 positive MVBs and lysosomes were distinguished by organelle morphology. (E) The percentage of OA1 positive organelles that were LAMP1 positive by fluorescence correlated well with the percentage of OA1 positive organelles that were morphologically identifiable as lysosomes by EM. Scale Bar:40µm and 200nm.

Supplementary Figure 3. OA1 colocalises well with PMEL and LBPA but not EGF. (A) HeLa cells expressing myc-tagged OA1 were labelled with anti-myc and anti-LBPA. (B) HeLa cells co-expressing myc-tagged OA1 and PMEL were incubated with EGF-488 for 25mins before labelling with anti-myc and anti-HMB45. (C) Colocalisation determined by the Mander's coefficient shows low colocalisation between EGF and OA1 whereas it is high for PMEL and LBPA.

Supplementary Figure 4. Expression of wild-type OA1 in HeLa cells reduces delivery of PMEL to Lyso Tracker positive lysosomes. HeLa cells transfected with either PMEL or a combination of PMEL and OA1-wt were incubated with Lyso Tracker for 30mins before chasing for 1hr. Cells expressing OA1-wt gave lower colocalisation between PMEL and Lyso Tracker, therefore indicating a restricted delivery of PMEL to lysosomes.

Supplementary Figure 1

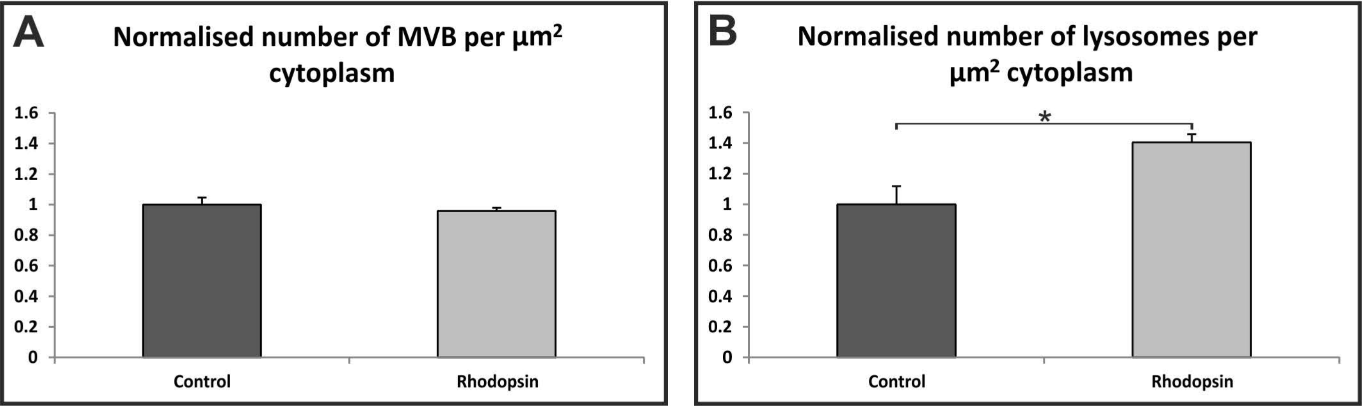

Supplementary Figure 2

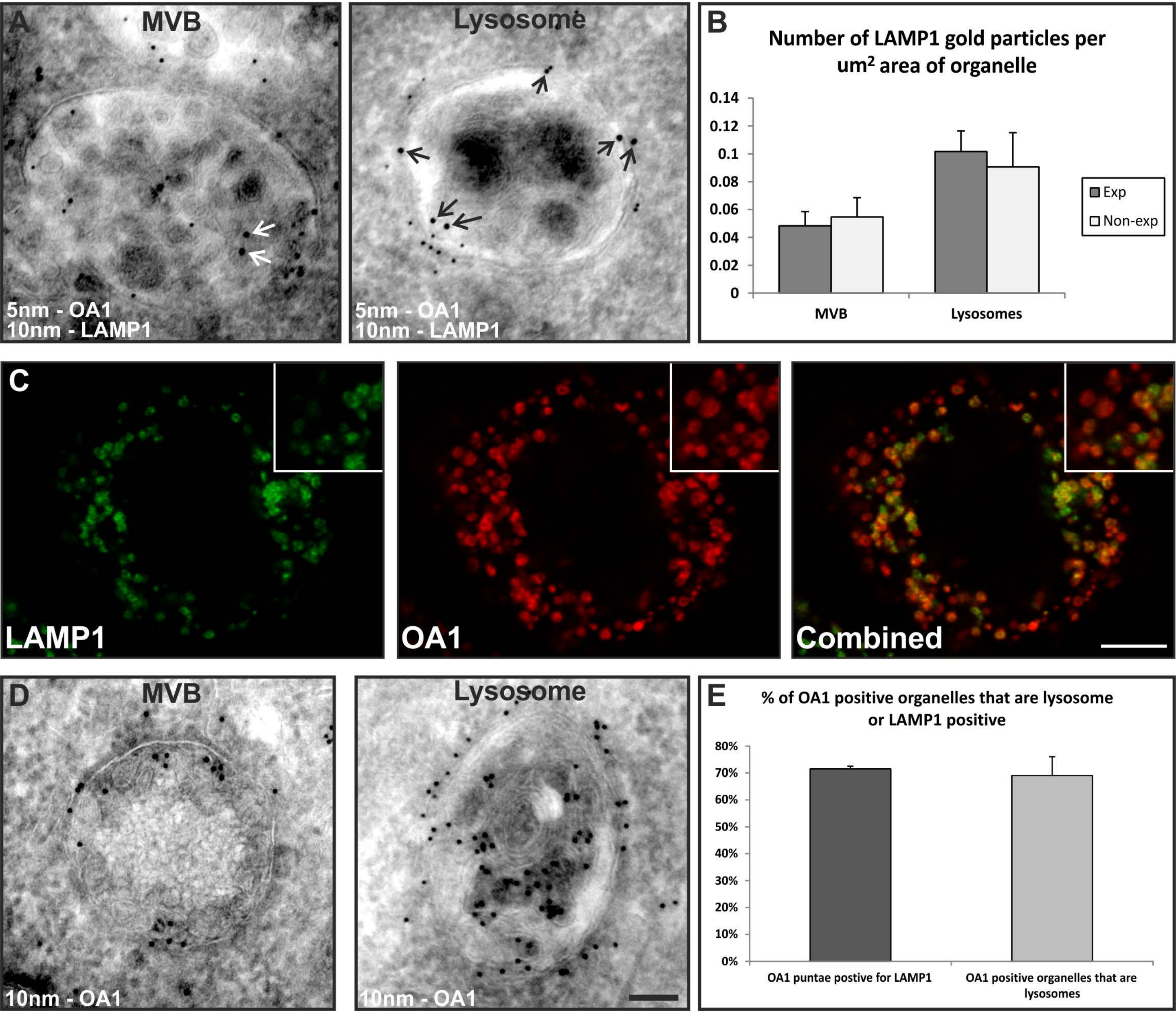

Supplementary Figure 3

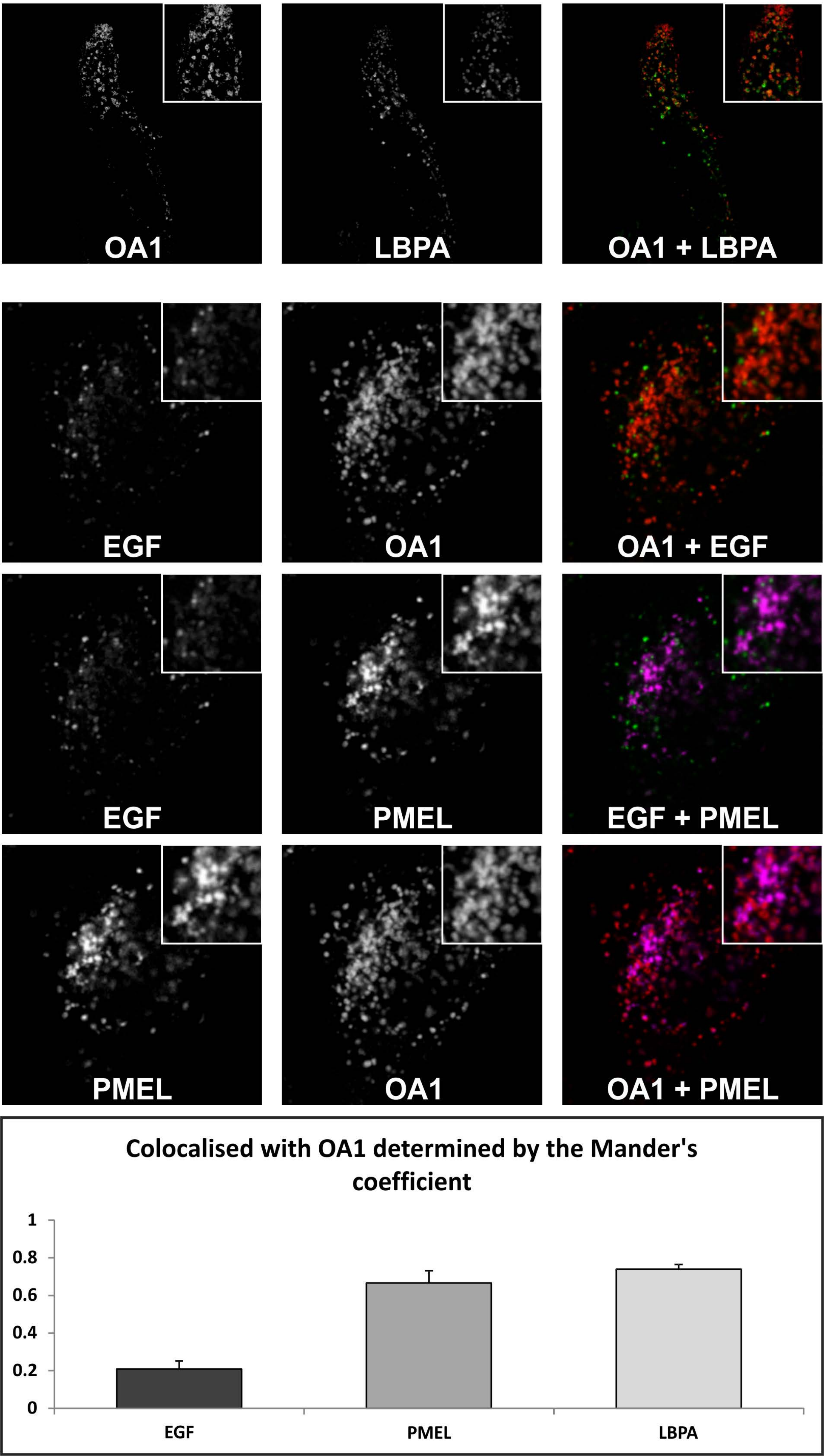

Supplementary Figure 4

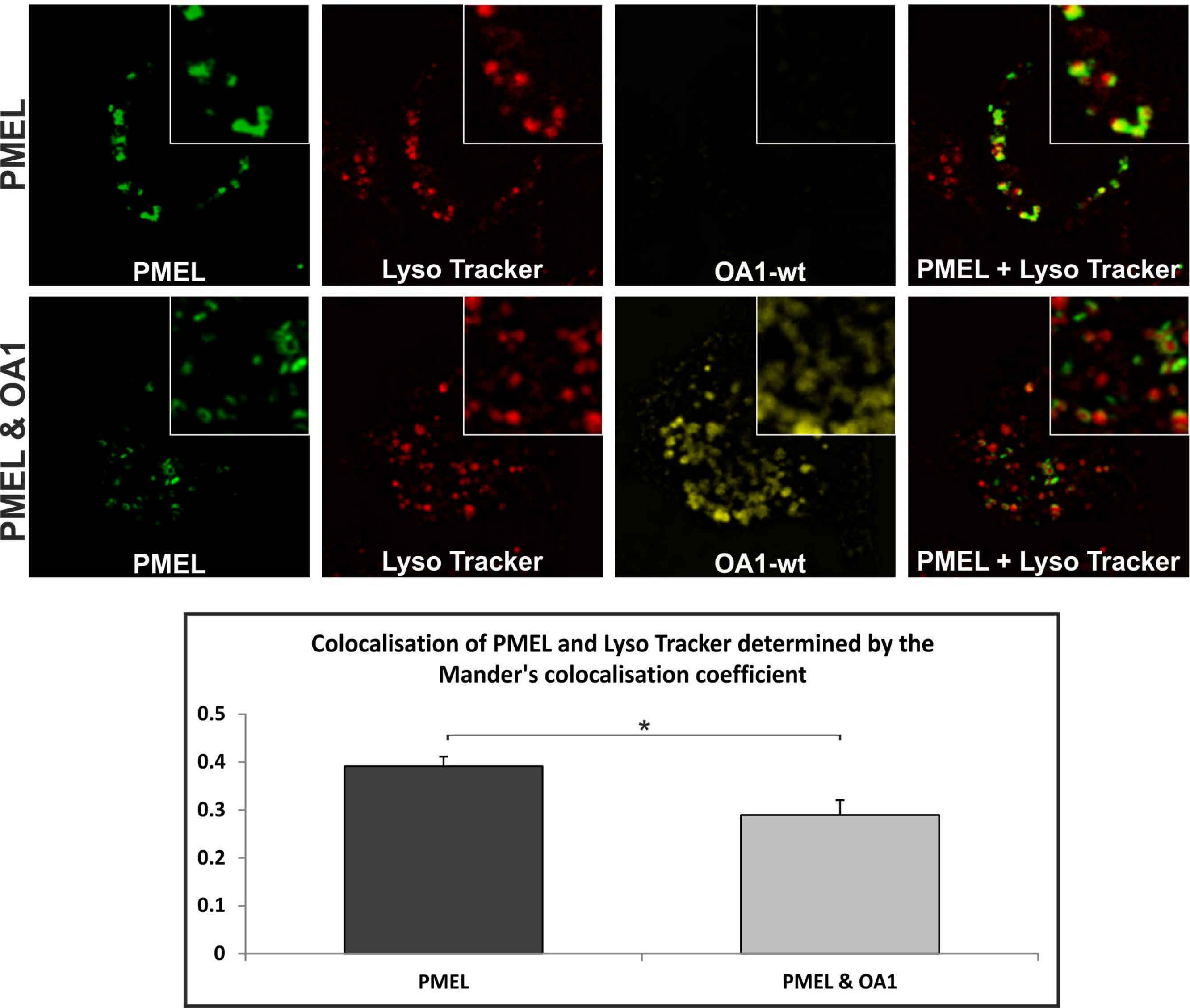

Supplement: Supplementary Material [file supp_126.22.5143_JCS128561.pdf]
